# Involvement of galanin and galanin receptor 2 in nociceptive modulation in anterior cingulate cortex of normal rats and rats with mononeuropathy

**DOI:** 10.1038/srep45930

**Published:** 2017-04-05

**Authors:** Meng-Lin Zhang, Hong-Bo Wang, Feng-Hua Fu, Long-Chuan Yu

**Affiliations:** 1 School of Pharmacy, Key Laboratory of Molecular Pharmacology and Drug Evaluation (Yantai University), Ministry of Education, Collaborative Innovation Center of Advanced Drug Delivery System and Biotech Drugs in Universities of Shandong, Yantai University, Yantai, 264005, P.R. China; 2 Neurobiology Laboratory, College of Life Sciences, Peking University, Beijing 100871, P R China

## Abstract

The present study was performed to explore the role of galanin and galanin receptor 2 in nociceptive modulation in anterior cingulate cortex (ACC) of normal rats and rats with mononeuropathy. Intra-ACC injection of galanin induced significant increases in hindpaw withdrawal latencies (HWLs) to thermal and mechanical stimulations in both normal rats and rats with mononeuropathy, the increased HWLs were attenuated significantly by intra-ACC injection of galanin receptor 2 antagonist M871, indicating an involvement of galanin receptor 2 in nociceptive modulation in ACC. Interestingly, the galanin-induced HWL was significant higher in rats with mononeuropathy than that in normal rats tested by Randall Selitto test. Furthermore, both the galanin mRNA expression and galanin content increased significantly in ACC in rats with mononeuropathy than that in normal rats. Moreover, both the mRNA levels of galanin receptor 2 and the content of galanin receptor 2 in ACC increased significantly in rats with mononeuropathy than that in normal rats. These results found that galanin induced antinociception in ACC in both normal rats and rats with mononeuropathy. And there may be plastic changes in the expression of galanin and galanin receptor 2 in rats with mononeuropathy, as well as in the galanin-induced antinociception.

The neuropeptide galanin, with 29 amino acids (30 in humans), is firstly isolated from porcine intestine^1^. It is well known that galanin is widely distributed in the central nervous system and peripheral nervous system^2^. There are three subtypes of galanin receptor (Gal R1, Gal R2 and Gal R3) and they belong to the family of G-protein coupled receptors^3^,^4^,^5^. Many studies have reported that galanin and its receptors are involved in many physiological functions and pathological processions^4^,^6^,^7^,^8^. Previous studies have demonstrated that galanin and galanin receptors play important roles in the transmission and/or modulation of nociception of the central nervous system, at spinal cord levels^7^,^9^, as well as in the brain, such as hypothalamic arcuate nucleus, nucleus accumbens, periaqueductal grey, and the central nucleus of amygdala^10^,^11^,^12^,^13^,^14^,^15^,^16^,^17^,^18^,^19^. Wang and her colleagues demonstrated that administration of galanin into periaqueductal grey induced significant antinociceptive effects in intact rats and rats with mononeuropathy^16^,^20^. Jin *et al*. reported that galanin induced antinociceptive effects in the central nucleus of amygdala of rats, and opioid receptors were involved in the galanin-induced antinociception^14^. Sun and his colleagues reported that intra-arcuate nucleus of hypothalamus administration of galanin induced significant antinociceptive effects in rats^15^. Recently, many studies have demonstrated that galanin receptor 2 (Gal R2) is involved in the modulation of neuropathic pain in the central nervous system^19^,^21^,^22^,^23^.

The anterior cingulate cortex (ACC) is one of the important structures of the brain^24^,^25^,^26^. Recently, many studies demonstrated that ACC is involved in the nociceptive modulation in the brain^25^,^26^,^27^,^28^,^29^,^30^,^31^,^32^. Neuropathic pain is one of common clinic pain^13^,^16^,^33^. Recently, many studies demonstrated that ACC is involved in the persistent neuropathic pain^25^,^28^,^31^. Activation of the ACC is associated with reduced pain behavior produced by unilateral spinal nerve injury in rats^34^. Study from Zhuo’s laboratory found that protein kinase M zeta (PKMζ) maintains pain-induced persistent changes in the mouse ACC, and microinjection of the selective PKMζ inhibitor into ACC blocked behavioral sensitization in mouse with peripheral nerve injury^28^.

The present study was performed to explore the role of galanin in nociceptive modulation in ACC of normal rats and rats with mononeuropathy, and further to explore the plastic changes in the expression of galanin and galanin receptor 2 in ACC in rats with mononeuropathy.

## Results

### Antinociceptive effects induced by intra-ACC administration of galanin in normal rats

**Figure 1:**
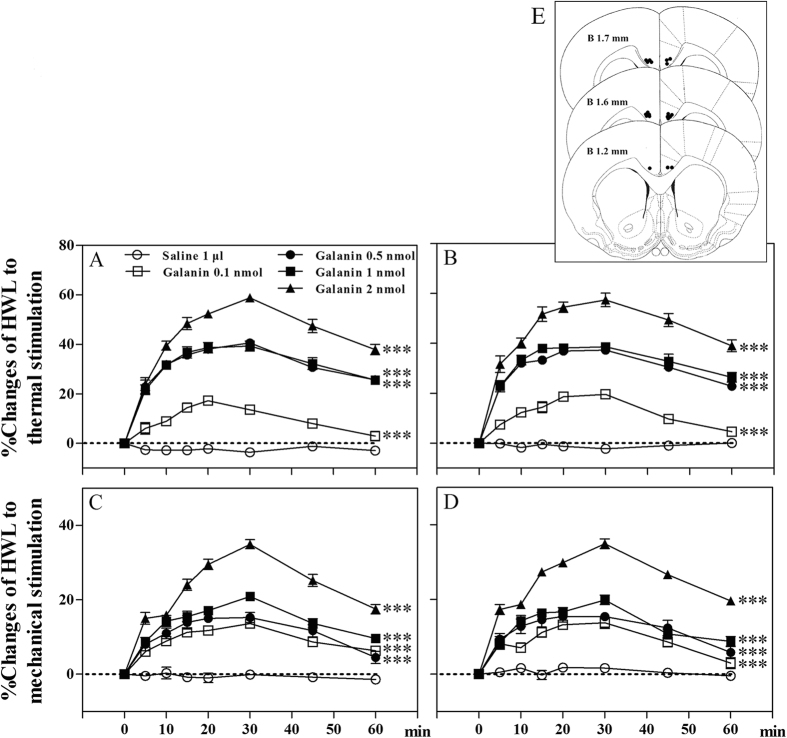
The HWLs to thermal (**A** and **B**) and mechanical stimulation (**C** and **D**) increased significantly after intra-ACC injection of galanin in normal rats. Left HWL: (**A** and **C**); right HWL: (**B** and **D**). Two-way ANOVA, ***P < 0.001. ACC, the anterior cingulate cortex; HWL, hindpaw withdrawal latency.

To determine the influence of galanin on nociceptive modulation in ACC of normal rats, five groups of rats received intra-ACC injection of 0.1 (n = 7), 0.5 (n = 8), 1 (n = 9) or 2 nmol (n = 8) of galanin, or 1 μl of 0.9% saline as a control (n = 10). The results are shown in Fig. 1.

As shown in Fig. 1, the hindpaw withdrawal latencies (HWLs) to thermal and mechanical stimulations increased significantly in a dose-dependent manner after intra-ACC injection of 0.1 nmol of galanin (Hot-plate Test: F_left/left_ = 84.27, P < 0.001; F_right/right_ = 98.05, P < 0.001; Randall Selitto Test: F_left/left_ = 193.10, P < 0.001; F_right/right_ = 57.21, P < 0.001), 0.5 nmol of galanin (Hot-plate Test: F_left/left_ = 797.30, P < 0.001; F_right/right_ = 834.90, P < 0.001; Randall Selitto Test: F_left/left_ = 84.59, P < 0.001; F_right/right_ = 60.43, P < 0.001), 1 nmol of galanin (Hot-plate Test: F_left/left_ = 414.10, P < 0.001; F_right/right_ = 394.90, P < 0.001; Randall Selitto Test: F_left/left_ = 387.20, P < 0.001; F_right/right_ = 179.40, P < 0.001) and 2 nmol of galanin (Hot-plate Test: F_left/left_ = 915.90, P < 0.001; F_right/right_ = 582.30, P < 0.001; Randall Selitto Test: F_left/left_ = 418.60, P < 0.001; F_right/right_ = 684.20, P < 0.001) compared with the control group. The results demonstrated that intra-ACC injection of galanin induced significant antinociceptive effects in normal rats.

At the end of the experiments, the location of the tip of the injection needle was verified and the injection points are shown in Fig. 1E. Only the results obtained from nociceptive tests that the tips of the injection needle are within the ACC are used for statistical analysis.

### Influences of intra-ACC injection of the galanin receptor 2 antagonist M871 on the galanin-induced antinociception in normal rats

To determine the involvement of galanin receptor 2 in galanin-induced antinociception in ACC of normal rats, four groups of rats received intra-ACC injection of 1 nmol of galanin, followed 5 min later by intra-ACC injection of 0.1 (n = 9), 0.5 (n = 9) or 1 nmol (n = 8) of the selective galanin receptor 2 antagonist M871, or 1 μl of 0.9% saline as a control (n = 7).

**Figure 2:**
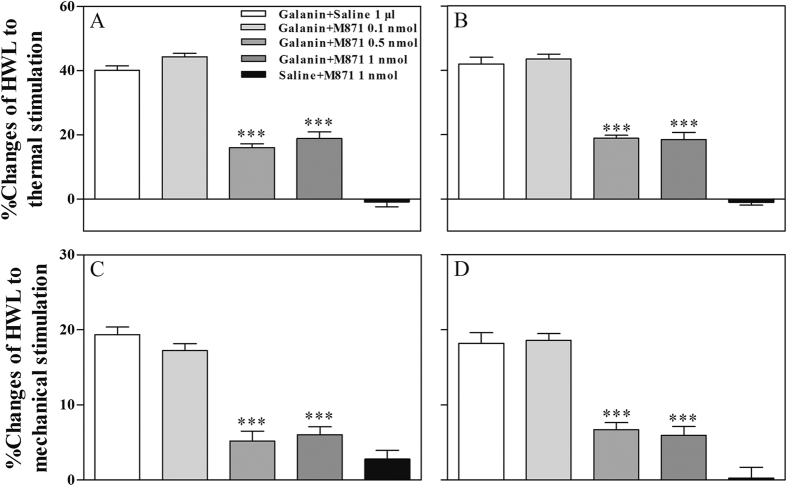
Effects of intra-ACC injection of M871 on the galanin-induced increases in HWLs to thermal (**A** and **B**) and mechanical stimulation (**C** and **D**) in normal rats. Left HWL: (**A** and **C**); right HWL: (**B** and **D**). One-way ANOVA, followed Dunnett multiple comparisons test, ***P < 0.001.

As shown in Fig. 2, compared with the control group, there were no significant influences on galanin-induced increases in HWLs after intra-ACC injection of 0.1 nmol of M871 (Hot-plate Test: Dunnett test, P = 0.13; Dunnett test, P = 0.85; Randall Selitto Test: Dunnett test, P = 0.41; Dunnett test, P = 0.99. One-way ANOVA, followed Dunnett multiple comparison test). The galanin-induced increases in HWLs were significantly attenuated after intra-ACC injection of 0.5 nmol of M871 (Hot-plate Test: Dunnett test, P < 0.001; Dunnett test, P < 0.001; Randall Selitto Test: Dunnett test, P < 0.001; Dunnett test, P < 0.001) and 1 nmol of M871 (Hot-plate Test: Dunnett test, P < 0.001; Dunnett test, P < 0.001; Randall Selitto Test: Dunnett test, P < 0.001; Dunnett test, P < 0.001) compared with the control group.

Another group of rats (n = 7) received intra-ACC injection of 1 μl of 0.9% saline, followed 5 min later by intra-ACC injection of 1 nmol of M871. The results demonstrated that there are no marked influences on the HWLs to thermal and mechanical stimulation after intra-ACC administration of the selective galanin receptor 2 antagonist M871, as shown in Fig. 2. These results indicate that galanin receptor 2 is involved in the galanin-induced antinociception in the ACC of normal rats.

### Antinociceptive effects induced by intra-ACC administration of galanin in rats with mononeuropathy

**Figure 3:**
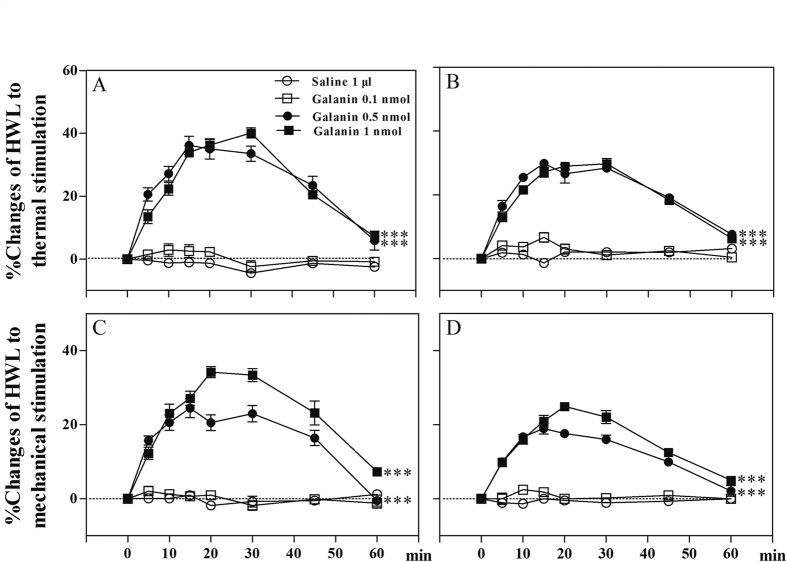
The HWLs to thermal (**A** and **B**) and mechanical stimulation (**C** and **D**) increased significantly after intra-ACC injection of galanin in rats with mononeuropathy. Left HWL: (**A** and **C**); right HWL: (**B** and **D**). Two-way ANOVA, ***P < 0.001.

In order to determine the influence of galanin on neuropathic pain, four groups of rats with sciatic nerve ligation received intra-ACC injection of 0.1 (n = 8), 0.5 (n = 8), or 1 nmol (n = 8) of galanin, or 1 μl of 0.9% saline as a control (n = 8), the results are shown in Fig. 3.

There were no significant increases in the HWLs to thermal and mechanical stimulations after intra-ACC injection of 0.1 nmol of galanin (Hot-plate Test: F_left/left_ = 4.32, P = 0.06; F_right/right_ = 2.54, P = 0.13; Randall Selitto Test: F_left/left_ = 0.08, P = 0.77; F_right/right_ = 3.21, P = 0.10) compared with control group in rats with mononeuropathy. The HWLs to thermal and mechanical stimulations increased significantly after intra-ACC injection of 0.5 nmol of galanin (Hot-plate Test: F_left/left_ = 162.50, P < 0.001; F_right/right_ = 286.00, P < 0.001; Randall Selitto Test: F_left/left_ = 85.52, P < 0.001; F_right/right_ = 161.90, P < 0.001), or 1 nmol of galanin (Hot-plate Test: F_left/left_ = 410.80, P < 0.001; F_right/right_ = 292.60, P < 0.001; Randall Selitto Test: F_left/left_ = 176.30, P < 0.001; F_right/right_ = 226.40, P < 0.001) compared with the control group in rats with mononeuropathy. The results demonstrated that intra-ACC injection of galanin induced significant antinociceptive effects in rats with mononeuropathy.

### Influences of intra-ACC injection of the galanin receptor 2 antagonist M871 on the galanin-induced antinociception in rats with mononeuropathy

**Figure 4:**
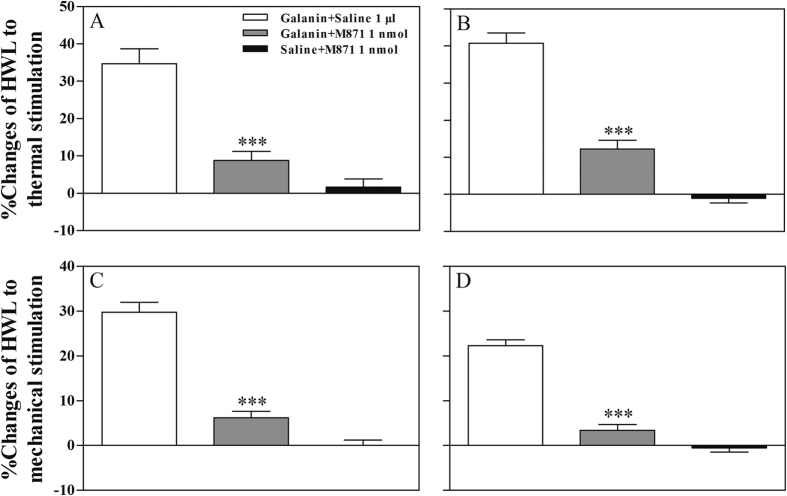
Effects of intra-ACC injection of M871 on the galanin-induced increases in HWLs to thermal (**A** and **B**) and mechanical stimulation (**C** and **D**) in rats with mononeuropathy. Left HWL: (**A** and **C**); right HWL: (**B** and **D**). Student’s t-test (two tails), ***P < 0.001.

Two groups of rats with mononeuropathy received intra-ACC injection of 1 nmol of galanin, followed 5 min later by intra-ACC injection of 1 nmol (n = 8) of the selective galanin receptor 2 antagonist M871, or 1 μl of 0.9% saline as a control (n = 7), the results are shown in Fig. 4.

After intra-ACC injection of 1 nmol of galanin, the HWL of rats with mononeuropathy increased markedly. The galanin-induced increases in HWLs were significantly attenuated after intra-ACC injection of 1 nmol of M871 (Hot-plate Test: t_left/left_ = 5.69, P < 0.001; t_right/right_ = 7.92, P < 0.001; Randall Selitto Test: t_left/left_ = 8.98, P < 0.001; t_right/right_ = 10.31, P < 0.001) compared with the control group.

Another group of rats with mononeuropathy (n = 8) received intra-ACC injection of 1 μl of 0.9% saline, followed 5 min later by intra-ACC injection of 1 nmol of M871, there were no marked influences on the HWLs to thermal and mechanical stimulation after intra-ACC administration of the selective galanin receptor 2 antagonist M871 in rats with mononeuropathy, as shown in Fig. 4.

### Comparison of the basal HWL and the galanin-induced antinociception in normal rats and rats with mononeuropathic pain

**Figure 5:**
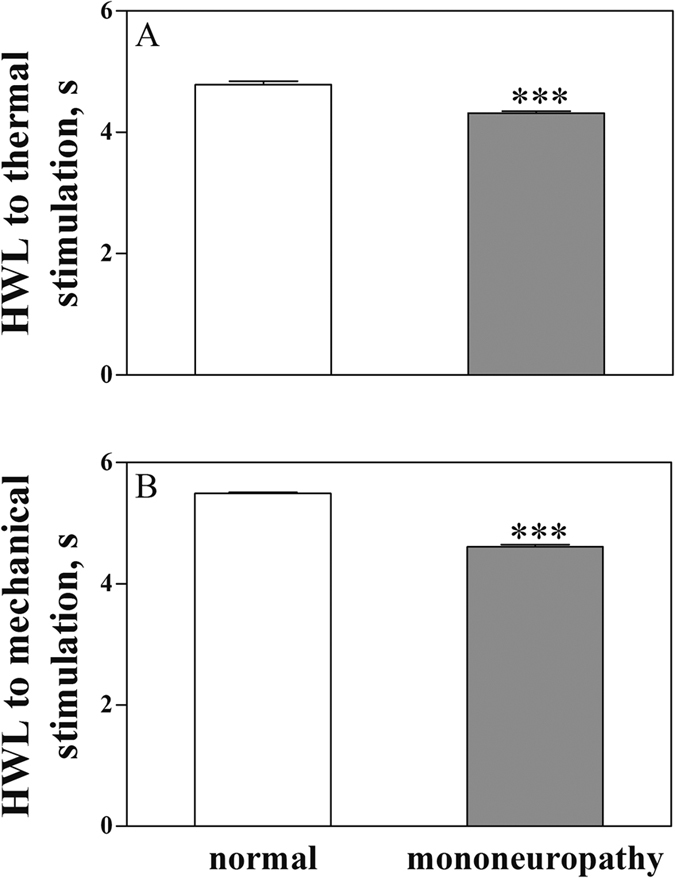
Comparison of the basal HWL in normal rats and rats with neuropathic pain. Left HWL: (**A** and **C**); right HWL: (**B** and **D**). Student’s t-test (two tails), ***P < 0.001.

The basal HWL in normal rats and rats with neuropathic pain are shown in Fig. 5. There were significant decreases in HWL to noxious thermal and mechanical stimulation in rats with neuropathic pain compared with normal rats (Hot-plate Test: t_left/left_ = 6.38, P < 0.001; Randall Selitto Test: t_left/left_ = 21.53, P < 0.001).

**Figure 6:**
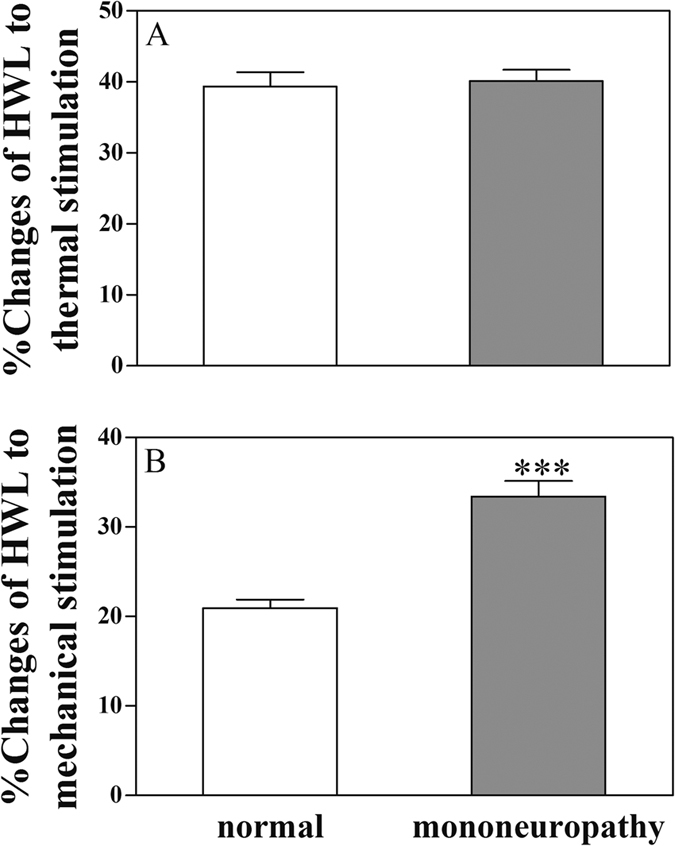
Comparison of the galanin-induced antinociception in normal rats and rats with neuropathic pain. Left HWL: (**A** and **C**); right HWL: (**B** and **D**). Student’s t-test (two tails), ***P < 0.001.

Figure 6 shows the changes in antinociceptive effects induced by intra-ACC injection of galanin in rats with neuropathic pain compared with that in normal rats. There was significant increase in HWL to noxious mechanical stimulation (Randall Selitto Test: t = 6.46, P < 0.001) in rats with neuropathic pain compared with normal rats as shown in Fig. 6B, well there was no significant increase in HWL to noxious thermal stimulation (Hot-plate Test: t = 0.29, P = 0.77) as shown in Fig. 6A.

### Comparison of the mRNA levels of galanin and the content of galanin in ACC of normal rats and rats with mononeuropathic pain

**Figure 7:**
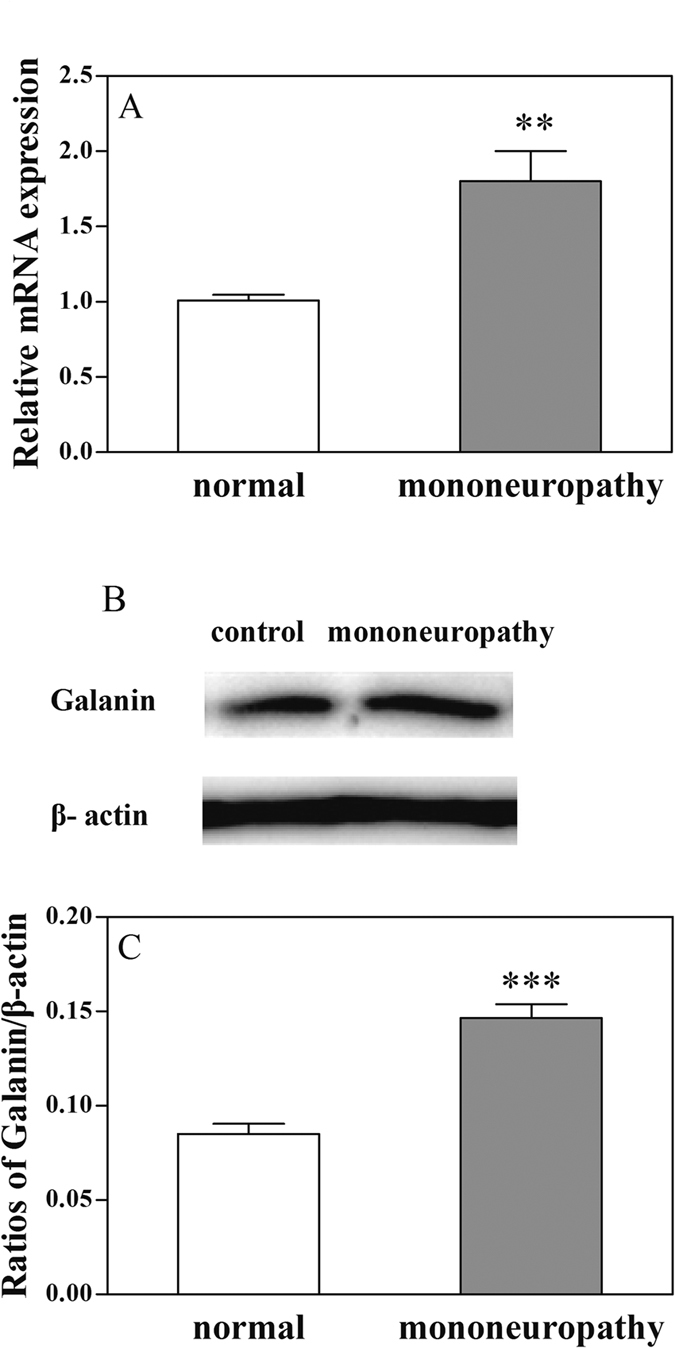
Changes in galanin mRNA levels and galanin contents in ACC of rats with left sciatic nerve ligation. (**A**) Results from RT-PCR test; (**B** and **C**) results from western blot test. Student’s t-test (two tails), **P < 0.01 and ***P < 0.001.

The galanin mRNA levels in ACC in normal rats and in rats with mononeuropathy were tested by real-time PCR (RT-PCR) and the results are shown in Fig. 7A. There was a significant increase in the mRNA level of galanin in ACC in rats with mononeuropathy than that in normal rats (t = 3.88, P < 0.01). The results indicate that the expression of galanin in ACC increased in rats with neuropathic pain.

We further determined the influence of sciatic nerve ligation-induce mononeuropathy on the content of galanin in ACC. The contents of galanin in ACC in normal rats and rats with mononeuropathy were tested by western blot and the results are shown in Fig. 7B and C. There was a significant increase in the content of galanin in ACC in rats with mononeuropathy (t = 6.83, P < 0.001) than that in normal rats.

The above results indicate that there may be increased changes of galanin expression in ACC induced by neuropathic pain in rats with sciatic nerve ligation.

### Comparison of the mRNA levels and the content of galanin receptor 2 in ACC in normal rats and rats with mononeuropathic pain

**Figure 8:**
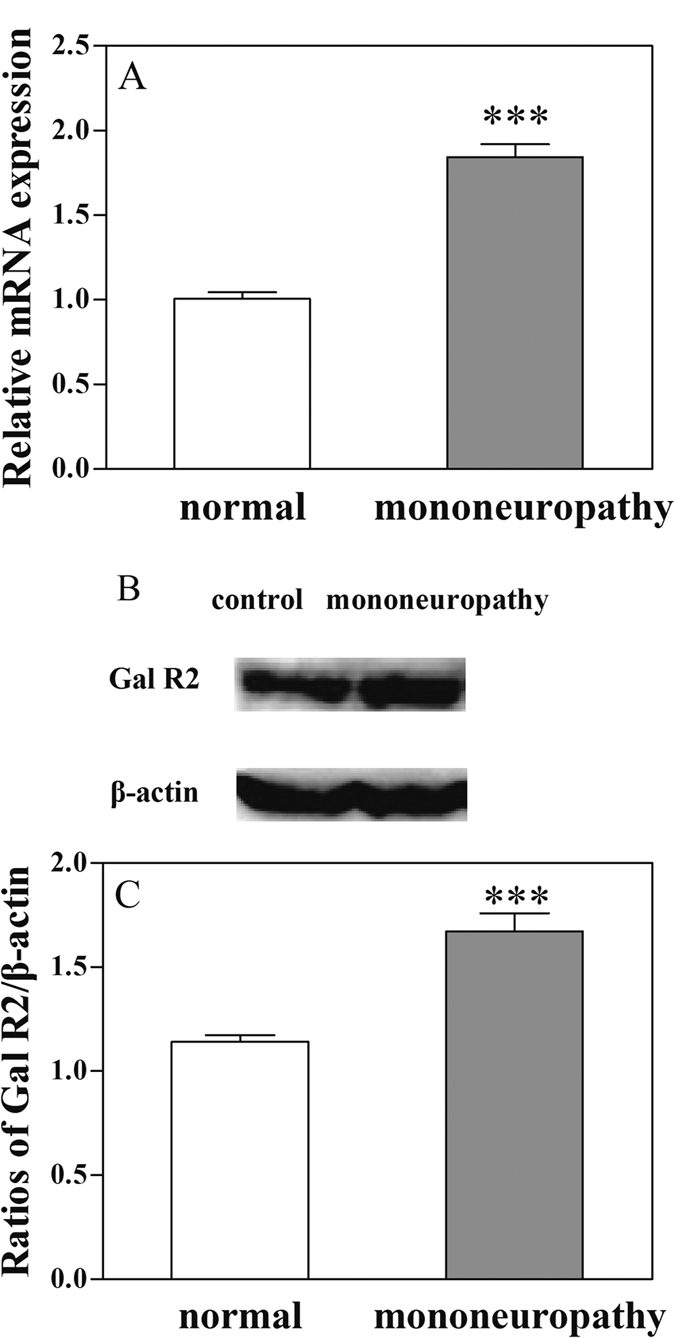
Changes in the mRNA level of galanin receptor 2 and galanin receptor 2 content in rats with left sciatic nerve ligation. (**A**) Results from RT-PCR test; (**B** and **C**), results from western blot test Galanin receptor 2, Gal R2. Student’s t-test (two tails), ***P < 0.001.

Our results have demonstrated that galanin receptor 2 is involved in the galanin-induced antinociception in the ACC in normal rats and rats with mononeuropathy. Here we try to find the changes of galanin receptor 2 induced by neuropathic pain in rats with sciatic nerve ligation. The mRNA levels of galanin receptor 2 in ACC and the content of galanin receptor 2 in ACC were determined by RT-PCR and western blot, and the results are shown in Fig. 8.

As shown in Fig. 8A, there was a significant increase in the mRNA level of galanin receptor 2 (t = 9.77, P < 0.001) in ACC in rats with mononeuropathy than that in normal rats tested by RT-PCR. The results indicate that the expression of galanin receptor 2 increased in ACC in rats with neuropathic pain.

Figure 8B and C show the changes of galanin receptor 2 content in ACC in rats with neuropathic pain. There was a significant increase in the content of galanin receptor 2 in ACC of rats with mononeuropathy (t = 5.80, P < 0.001) than that in normal rats tested by western blot. The above results indicate that there may be plastic changes in the expression of galanin receptor 2 in ACC in rats with mononeuropathy.

## Discussion

The present study demonstrated that administration of galanin into ACC induced significant increases in HWLs to noxious thermal and mechanical stimulations in normal rats and rats with mononeuropathy, indicate that galanin is involved in pain modulation in ACC in both normal rats and rats with mononeuropathy. Consistent with our findings, administration of galanin to the midbrain periaqueductal grey induced significant antinociceptive effects in intact rats and rats with mononeuropathy^16^,^20^. Jin and his colleagues reported that galanin induced antinociceptive effects in the central nucleus of amygdala of rats, and opioid receptors are involved in the galanin-induced antinociception^14^.

There are few reports related to the roles of galanin receptor 2 in the brain in pain modulation. Some studies showed that there were no marked differences in pain related behaviors between the mice lacking galanin receptor 2 and WT mice^35^. Interestingly, while mice with an absence of galanin receptor 2 gene transcription displayed significant deficits in pain behavior in neuropathic and inflammatory pain models^36^. Recent studies have demonstrated that galanin receptor 2 is involved in neuropathic pain^5^,^21^,^23^. Hulse and his collogues reported that the high level of endogenous galanin in injured primary afferents activate peripheral galanin receptor 2, which leads to an increase in c-fibre mechanical activation thresholds and a marked reduction in evoked and ongoing nociceptive responses^21^. Recently, Zhang and her colleagues reported that galanin receptor 2 is involved in the galanin-induced antinociceptive effects in the periaqueductal grey of rats^19^. As galanin receptor 2 may play an important role in nociceptive modulation in the central nervous system, the present study was further to explore the role of galanin receptor 2 in pain modulation in ACC of rats and found that the selective galanin receptor 2 antagonist M871 attenuated the galanin-induced antinociceptive effects in ACC, suggesting that galanin receptor 2 is involved in the galanin-induced antinociceptive effects in ACC in both normal rats and rats with mononeuropathy.

Recently, studies demonstrated that ACC is involved in the modulation of nociception^25^,^31^,^37^. Activation of the ACC is associated with reduced pain behavior produced by unilateral spinal nerve injury in rats^34^. The ACC is also reported to be involved in the persistent neuropathic pain^31^,^38^,^39^. The nerve cells in ACC responded to noxious stimuli and were activated during pain tested by electrophysiological methods^40^. In the present study, we found that intra-ACC injection of galanin induced dose-dependent increases in the latencies of hindpaw withdrawal to noxious thermal and mechanical stimulations in rats, suggesting that galanin induces antinociceptive effects in the ACC.

Chronic pain induced plasticity in central nervous system has been paid more and more attention. Recently Zhuo and his colleagues reviewed the synaptic plasticity in the anterior cingulate cortex in acute and chronic pain. In this review, they discussed that ACC activation contributes to chronic pain states and describe several forms of synaptic plasticity that may underlie the effect, in particular, one form of long-term potentiation (LTP) in the ACC sustains the affective component of the pain state^41^. In the present study, we found that both the galanin mRNA expression and galanin content increased significantly in ACC in rats with mononeuropathy than that in normal rats. Moreover, both the mRNA level of galanin receptor 2 and the content of galanin receptor 2 in ACC increased significantly in rats with mononeuropathy than that in normal rats. Combining the behavioral tests with these results indicate that there may be plasticity in the expression of galanin and galanin receptor 2 in rats with mononeuropathy, as well as in the galanin-induced antinociception.

Taken together, the present study found for the first time that galanin induces antinociception in ACC in both normal rats and rats with mononeuropathy. And there may be plastic changes in the expression of galanin and galanin receptor 2 in rats with mononeuropathy, as well as in the galanin-induced antinociception.

## Methods

### Chemicals

Solutions for intra-ACC administration were prepared with sterilized saline, each with a volume of 1 μl containing: (1) 0.1, 0.5, 1, or 2 nmol of galanin (rat galanin, 1–29 amino acids, Tocris, UK); (2) 0.1, 0.5, or 1 nmol of M871 (selective galanin receptor 2 antagonist; Tocris, UK).

### Animal preparation

All experiments were carried out on freely moving male Sprague–Dawley rats weighing 220 to 260 g (Experimental Animal Center of Luye Pharmaceutical Company, Yantai, China). The rats were housed in cages with free access to food and water, and maintained in a room temperature of 20 ± 2 °C with a normal day/night cycle. All experiments were performed according to the guidelines of the International Association for the Study of Pain^42^ and The Guidelines for the Care and Use of Laboratory Animals of Yantai University. Our experiments were approved by Laboratory Animal Ethics Committee of Yantai University and the authorization number is 20140901-01. And every effort was made to minimize both the animal suffering and the number of animals used.

### Nociceptive tests

Rats were accustomed to the test condition for 3 days before the experiment to minimize the stress induced by handling and measurements. The hindpaw withdrawal latencies (HWLs) during thermal and mechanical stimulation were measured as described previously^14^,^15^,^43^. Briefly, the entire ventral surface of the rat hindpaw was placed manually on a hot plate (YLS-6B Intelligent Heat Panel Instrument, Jinan Yiyan Science & Technology Development Co., Ltd., Jinan, China), which was maintained at a temperature of 52 ± 0.2 °C. The time to hindpaw withdrawal was measured in seconds and referred to as the HWL to thermal stimulation. The Randall Selitto Test (Ugo Basile, Type 7200, Italy) was used to assess the HWL to mechanical stimulation. A wedge-shaped pusher at a loading rate of 30 g/s was applied to the dorsal surface of the hindpaw. The latency required to initiate the withdrawal response was assessed and expressed in seconds. Before intra-ACC injection, the HWLs were tested three times and regarded as the basal HWLs. The HWLs recorded during subsequent experiments were expressed as percentage changes of the basal level for each rat (% changes of the HWL). Each rat was tested by both types of stimulation. Every measurement of the HWL to both thermal and mechanical stimulation was finished within 1–2 min. A cut-off limit of 15s was set up to avoid tissue damage.

### Surgical procedures and intra-ACC injection

Rats were anaesthetized by intraperitoneal injection of pentobarbital sodium (50 mg/kg, Xudong Chemical Factory, Beijing, China) and mounted on stereotaxic frame, a stainless steel guide cannula of 0.8 mm outer diameter was directed to the ACC (1.6 mm anterior to Bregma; 0.7 mm lateral to midline; 2.0 mm ventral to the surface of skull) according to Paxinos and Watson^44^, and was fixed to the skull by dental acrylic. There were more than 3 days for rats to recover from the operation. On the day of experiment, a stainless steel needle with 0.4 mm outer diameter was directly inserted into the guide cannula with 1.5 mm beyond the tip of the latter. One microliter of solution was thereafter infused into the ACC over 1 min. The injection needle was left in the site for 1 min after each injection before removal.

### The rat model of mononeuropathy

The mononeuropathy model was produced as previously reported^13^,^43^. Animals were anaesthetized by intraperitoneal injection of pentobarbital sodium (50 mg/kg) and 8–10 mm of the left common sciatic nerve was exposed at the level of the mid-high. Four loose ligatures were placed around the dissected nerve at 1.0–1.5 mm intervals. The ligations were carefully manipulated so that the nerve was barely constricted, and the skin incision was closed with silk sutures and animals allowed to recover.

### RT-PCR

Normal rats (n = 3, as a control) and rats with sciatic nerve ligation 10 days after surgery (n = 3) received injection of over dose of 10% trichloroacetaldehyde monohydrate and the brain was removed immediately. The regions of the ACC were dissected on ice and the total RNA was isolated with UNIQ-10 Column Trizol Total RNA Extraction Kit (Sangon Biotech, Shanghai, China) following the instruction. Total RNA was firstly reverse-transcribed using AMV First Strand cDNA Synthesis Kit (Sangon Biotech, Shanghai, China), and the RT-PCRs were performed using StepOne™ Real-Time PCR System and software (Thermo Fisher Scientific, USA). Sequences of primers for the experiments were rat galanin: sense 5′-CACATGCCATTGACAACCAC-3′ and antisense 5′-AACTCCATTATAGTGCGGACG-3′; rat galanin receptor 2: sense 5′-GCCGCCATCGGGCTCATCTG-3′ and antisense 5′-GTCGAGGTGCGCTCCATGCT-3′); rat GAPDH: sense 5′-GACCACCCAGCCCAGCAAGG-3′and antisense 5′-TCCCCAGGCCCCTCCTGTTG-3′. The unigene expression levels were calculated with the 2(-delta delta C (T)) method^45^.

### Western Blot

The normal rats (n = 3, as a control) and rats with sciatic nerve ligation 10 days after surgery (n = 3) received injection of over dose of 10% trichloroacetaldehyde monohydrate and the brain was removed immediately. The regions of the ACC were dissected on ice and then frozen at −80 °C. Total protein was extracted following our published protocol^46^, briefly the ACC samples homogenized in RIPA lysis buffer (Beyotime Institute of Biotechnology, Shanghai, China), the total protein was extracted and the whole protein extracts (80 μg) of ACC samples were subject to western blot assay. After transferred to PVDF membranes (Millipore, MA, USA), the membranes were incubated in blocking solution and then incubated with the polyclonal goat anti-galanin antibody (SAB2501407, Sigma–Aldrich, St. Louis, MO, USA), polyclonal rabbit anti-Gal R2 antibody (ab203072, Abcam, Cambridge, UK), beta-actin antibody (Beyotime Institute of Biotechnology, Shanghai, China) at 4 °C overnight. The membranes were washed with TBST and then probed with HRP-conjugated donkey anti-goat secondary antibody (Beyotime Institute of Biotechnology, Shanghai, China), HRP-conjugated goat anti-rabbit secondary antibody (Beyotime Institute of Biotechnology, Shanghai, China), HRP-conjugated goat anti-mouse secondary antibody (Beyotime Institute of Biotechnology, Shanghai, China). The brands were visualized by enhanced chemiluminescence (ECL) detection reagents (Beyotime Institute of Biotechnology, Shanghai, China) and imaged using ImageQuant LAS 4000 (GE Healthcare Bio-Sciences AB, Tokyo, Japan) automatically and quantified using ImageQuant software (GE Healthcare Bio-Sciences AB, Tokyo, Japan).

### Statistical analysis

At the end of the experiments, the location of the tip of the injection needle was verified and the injection points were in ACC of rats. Only the results obtained from nociceptive tests that the tips of the injection needle are within the ACC are used for statistical analysis. Data from the experiment were expressed as mean ± S.E.M. Statistical difference between groups was determined by Two-way analysis of variance (ANOVA) for repeated measurements (F_left/left_ is the F value of the two groups: the left HWL of one group compared with the left HWL of another group), One-way ANOVA followed Dunnett multiple comparison test or by Student’s t test (two-tailed). *P < 0.05, **P < 0.01 and ***P < 0.001 were considered as significant differences.

## Additional Information

**How to cite this article:** Zhang, M.-L. *et al*. Involvement of galanin and galanin receptor 2 in nociceptive modulation in anterior cingulate cortex of normal rats and rats with mononeuropathy. *Sci. Rep.* **7**, 45930; doi: 10.1038/srep45930 (2017).

**Publisher's note:** Springer Nature remains neutral with regard to jurisdictional claims in published maps and institutional affiliations.

## References

[b1] Tatemoto K., Rokaeus A., Jornvall H., Mcdonald T. J. & Mutt V. Galanin—a novel biologically active peptide from porcine intestine. FEBS Letters. 164, 124–128 (1983). 6197320 10.1016/0014-5793(83)80033-7

[b2] Melander T., Hokfelt T. & Rokaeus A. Distribution of galanin-like immunoreactivity in the rat central nervous system. J. Comp. Neurol. 248, 475–517 (1986). 2424949 10.1002/cne.902480404

[b3] Branchek T. A., Smith K. E., Gerald C. & Walker M. W. Galanin receptor subtypes. Trends Pharmacol. Sci. 21, 109–117 (2000). 10689365 10.1016/s0165-6147(00)01446-2

[b4] Lang R., Gundlach A. & Kofler B. The galanin peptide family: receptor pharmacology, pleiotropic biological actions, and implications in health and disease. Pharmacol. Ther. 115, 177–207 (2007). 17604107 10.1016/j.pharmthera.2007.05.009

[b5] Mitsukawa K., Lu X. & Bartfai T. Galanin, galanin receptors and drug targets. Cell. Mol. Life Sci. 65, 1796–1805 (2008). 18500647 10.1007/s00018-008-8153-8 PMC11131746

[b6] Cheng Y. & Yu L.-C. Galanin protects amyloid-β-induced neurotoxicity on hippocampal neurons in rats. J. Alzheimer’s Dis. 20, 1143–1157 (2010). 20413891 10.3233/JAD-2010-091234

[b7] Hokfelt T. & Tatemoto K. Galanin–25 years with a multitalented neuropeptide. Cell Mol. Life Sci. 65, 1793–1795 (2008). 18500648 10.1007/s00018-008-8152-9 PMC11131681

[b8] Walton K. M., Chin J. E., Duplantier A. J. & Mather R. J. Galanin function in the central nervous system. Curr. Opin. Drug Disc. 9, 560–570 (2006). 17002216

[b9] Xu X. . Effects of exogenous galanin on neuropathicpain state and change of galanin and its receptors in DRG and SDH after sciatic nerve-pinch injury in rat. PLoS One 7, e37621 (2012). 22624057 10.1371/journal.pone.0037621 PMC3356287

[b10] Amorim D. . A role of supraspinal galanin in behavioural hyperalgesia in the rat. PLoS One 9, e113077 (2014). 25405608 10.1371/journal.pone.0113077 PMC4236133

[b11] Amorim D. . Galanin-mediated behavioural hyperalgesia from the dorsomedial nucleus of the hypothalamus involves two independent descending pronociceptive pathways. PLoS One 10, e0142919 (2015). 26565961 10.1371/journal.pone.0142919 PMC4643915

[b12] Fu L. B., Wang X. B., Shi J., Wu X. & Yu L.-C. Antinociceptive effects of intracerebroventricular injection of the galanin receptor 1 agonist M 617 in rats. Neurosci. Lett. 491, 174–176 (2011). 21241771 10.1016/j.neulet.2011.01.030

[b13] Gu X. L., Sun Y. G. & Yu L.-C. Involvement of galanin in nociceptive regulation in the arcuate nucleus of hypothalamus in rats with mononeuropathy. Behav Brain Res. 179, 331–335 (2007). 17383023 10.1016/j.bbr.2007.02.033

[b14] Jin W. Y., Liu Z., Liu D. & Yu L.-C. Antinociceptive effects of galanin in the central nucleus of amygdala of rats, an involvement of opioid receptors. Brain Res. 1320, 16–21 (2010). 20051236 10.1016/j.brainres.2009.12.060

[b15] Sun Y., Gu X., Lundeberg T. & Yu L.-C. An antinociceptive role of galanin in the arcuate nucleus of hypothalamus in intact rats and rats with inflammation. Pain 106, 143–150 (2003). 14581121 10.1016/s0304-3959(03)00316-6

[b16] Wang D., Lundeberg T. & Yu L.-C. Antinociceptive Role of galanin in periaqueductal grey of rats with experimentally induced monomononeuropathy. Neuroscience 96, 767–771 (2000). 10727794 10.1016/s0306-4522(00)00005-1

[b17] Wu X. & Yu L.-C. Alternation of galanin in nociceptive modulation in the central nervous system of rats during morphine tolerance: a behavioral and immunohistochemical study. Brain Res. 1086, 85–91 (2006). 16626663 10.1016/j.brainres.2005.12.132

[b18] Xu S. L., Li J., Zhang J. J. & Yu L. C. Antinociceptive effects of galanin in the nucleus accumbens of rats. Neurosci. Lett. 520, 43–46 (2012). 22595465 10.1016/j.neulet.2012.05.027

[b19] Zhang X. Y., Zhang Y. M., Zhang M. L. & Yu L. C. Involvement of galanin receptor 2 and CaMKII in galanin-induced antinociception in periaqueductal grey of rats. Neurosci. Lett. 604, 124–127 (2015). 26254694 10.1016/j.neulet.2015.08.005

[b20] Wang D., Ye H., Yu L.-C. & Lundeberg T. Intra-periaqueductal grey injection of galanin increases the nociceptive response latency in rats, an effect reversed by naloxone. Brain Res. 834, 152–154 (1999). 10407105 10.1016/s0006-8993(99)01513-9

[b21] Hulse R. P., Wynick D. & Donaldson L. F. Activation of the galanin receptor 2 in the periphery reverses nerve injury-induced allodynia. Mol. Pain 7, 26 (2011). 21496293 10.1186/1744-8069-7-26 PMC3101129

[b22] Hulse R. P., Donaldson L. F. & Wynick D. Peripheral galanin receptor 2 as a target for the modulation of pain. Pain Res. Treat. 2012, 545386(2012). 10.1155/2012/545386 PMC3270467 22315681

[b23] Metcalf C. S. . Analgesic properties of a peripherally acting and GalR2 receptor–preferring galanin analog in inflammatory, neuropathic, and acute pain models. J. Pharmacol. Exp. Ther. 352, 185–193 (2015). 25347995 10.1124/jpet.114.219063 PMC4279104

[b24] Devinsky O., Morrell M. J. & Vogt B. A. Contributions of anterior cingulate cortex to behaviour. Brain 118, 279–306 (1995). 7895011 10.1093/brain/118.1.279

[b25] Shen F.-Y. . Alleviation of neuropathic pain by regulating T-type calcium channels in rat anterior cingulated cortex. Mol. Pain 11, 7, doi: 10.1186/s12990-015-0008-3 (2015). 25885031 PMC4357203

[b26] Zhuo M. Contribution of synaptic plasticity in the insular cortex to chronic pain. Neuroscience 338, 220–229 (2016). 27530697 10.1016/j.neuroscience.2016.08.014

[b27] Gu L. . Pain inhibition by optogenetic activation of specific anterior cingulate cortical neurons. PLoS ONE 10, e0117746, doi: 10.1371 (2015). 25714399 10.1371/journal.pone.0117746 PMC4340873

[b28] Li X. Y. . Alleviating neuropathic pain hypersensitivity by inhibiting PKMzeta in the anterior cingulate cortex. Science 330, 1400–1404 (2010). 21127255 10.1126/science.1191792

[b29] Lu B. . Inhibition of mammalian target of rapamycin activation in the rostral anterior cingulate cortex attenuates pain-related aversion in rats. Behav. Brain Res. 310, 51–58 (2016). 27163752 10.1016/j.bbr.2016.05.011

[b30] Lieberman M. D. & Eisenberger N. I. The dorsal anterior cingulate cortex is selective for pain: Results from large-scale reverse inference. PANS. 112, 15250–15255 (2015). 10.1073/pnas.1515083112 PMC4679028 26582792

[b31] Xu H. . Presynaptic and postsynaptic amplifications of neuropathic pain in the anterior cingulated cortex. J. Neurosci. 28, 7445–7453 (2008). 18632948 10.1523/JNEUROSCI.1812-08.2008 PMC3844787

[b32] Zhuo M. Multiple PKCε-dependent mechanisms mediating mechanical hyperalgesia. Pain 150, 17–21 (2010). 20456866 10.1016/j.pain.2010.02.011 PMC2916056

[b33] Brian C. . Diabetic mononeuropathy: Clinical manifestations and current treatments. Lancet Neurol. 11, 521–534 (2012). 22608666 10.1016/S1474-4422(12)70065-0 PMC4254767

[b34] LaBuda C. J. & Fuchs P. N. Attenuation of negative pain affect produced by unilateral spinal nerve injury in the rat following anterior cingulate cortex activation. Neuroscience 136, 311–322 (2005). 16404776 10.1016/j.neuroscience.2005.07.010

[b35] Shi T. J. . Sensory neuronal phenotype in galanin receptor 2 knockout mice: focus on dorsal root ganglion neuron development and pain behaviour. Eur. J. Neurosci. 23, 627–36 (2006). 16487144 10.1111/j.1460-9568.2006.04593.x

[b36] Hobson S. A., Holmes F. E., Kerr N. C., Pope R. J. & Wynick D. Mice deficient for galanin receptor 2 have decreased neurite outgrowth from adult sensory neurons and impaired pain-like behaviour. J. Neurochem. 99, 1000–1010 (2006). 17076662 10.1111/j.1471-4159.2006.04143.x PMC2725756

[b37] Zhuo M. Molecular mechanisms of pain in the anterior cingulate cortex. J. Neurosci. Res. 84, 927–933 (2006). 16862566 10.1002/jnr.21003

[b38] Widerström-Noga E. . Metabolite concentrations in the anterior cingulate cortex predict high neuropathic pain impact after spinal cord injury. Pain 154, 204–212 (2013). 23141478 10.1016/j.pain.2012.07.022 PMC3670594

[b39] Zhang L. . Brain-derived neurotrophic factor (BDNF) in the rostral anterior cingulate cortex (rACC) contributes to neuropathic spontaneous pain-related aversion via NR2B receptors. Brain Res. Bull. 127, 56–65 (2016). 27575004 10.1016/j.brainresbull.2016.08.016

[b40] Yamamura H. . Morphological and electrophysiological properties of ACC nociceptive neurons in rats. Brain Res. 735, 83–92 (1996). 8905172 10.1016/0006-8993(96)00561-6

[b41] Bliss T. V., Collingridge G. L., Kaang B. K. & Zhuo M. Synaptic plasticity in the anterior cingulate cortex in acute and chronic pain. Nat. Rev. Neurosci. 17, 485–96 (2016). 27307118 10.1038/nrn.2016.68

[b42] Zimmermann M. Ethical guidelines for investigations of experimental pain in conscious animals. Pain 16, 109–10 (1983). 6877845 10.1016/0304-3959(83)90201-4

[b43] Bian H. & Yu L. C. Intra-nucleus accumbens administration of the calcium/calmodulin-dependent protein kinase II inhibitor AIP induced antinociception in rats with monomononeuropathy. Neurosci. Lett. 599, 129–132 (2015). 26022629 10.1016/j.neulet.2015.05.048

[b44] Paxinos G. & Watson C. The rat brain in stereotaxic coordinates, fourth ed., (Academic Press, Sydney 1998).

[b45] Livak K. J. & Schmittgen T. D. Analysis of relative gene expression data using real-time quantitative PCR and the 2(-delta delta C (T)) method. Methods. 25, 402–408 (2001). 11846609 10.1006/meth.2001.1262

[b46] Li M. . Oral administration of escin inhibits acute inflammation and reduces intestinal mucosal injury in animal models. EVID-BASED COMPL ALT 2015, Article ID 503617, 9 pages (2015). 10.1155/2015/503617 PMC4496496 26199634

